# Dynamic Dossier in the Cloud: A Sociotechnical Architecture for a Real-Time and Metrics-Based Data Tracking System with Gene and Cell Therapies as a Case Study

**DOI:** 10.1007/s43441-020-00227-y

**Published:** 2020-10-28

**Authors:** Kevin Nam, Kay Larholt, Gigi Hirsch, Paul Beninger, David Fritsche, Diane Shoda, John Ferguson, Florence T. Bourgeois, Donna Palmer, Karen Katz, Matt W. Courtney

**Affiliations:** 1 MIT Lincoln Laboratory, Massachusetts Institute of Technology, 244 Wood St., Lexington, MA 02421 USA; 2 MIT Center for Biomedical Innovation, Massachusetts Institute of Technology, 77 Massachusetts Avenue, Building E19-604, Cambridge, MA 02139 USA; 3 Public Health & Community Medicine, Tufts University School of Medicine, Boston, USA; 4 Greyscaling LLC, 3722 Las Vegas Blvd S, Unit 2111, Las Vegas, NV 89158 USA; 5 Pharmacovigilance Specialty Care, Sanofi-Genzyme Business Unit, 50 Binney St., Cambridge, MA 02142 USA; 6 Harvard Medical School, 300 Longwood Avenue Boston, Boston, MA 02115 USA; 7 FoCUS Project, NEWDIGS, MIT Center for Biomedical Innovation, Massachusetts Institute of Technology, 77 Massachusetts Avenue, Building E19, Cambridge, MA 02139 USA

**Keywords:** Multi-stakeholder sharing, Systems engineering, Gene and cell therapies, Scalable data architecture, Payers, Regulatory agencies

## Abstract

**Background:**

Data sharing among stakeholders in the development, access, and use of drug therapies is critical but the current system and process are inefficient.

**Methods:**

We take a Systems Engineering approach with a realistic use case to propose a scalable design for multi-stakeholder data sharing.

**Results:**

We make three major contributions to the drug development and healthcare communities: first, a methodology for developing a multi-stakeholder data sharing system, with its focus on high-level requirements that influence the design of the system architecture and technology choice; second, the development of a realistic use case for long-term patient and therapy data tracking and sharing in the use of potentially curative and durable gene and cell therapies. Further, a bridge for the ‘awareness gap’ was found between the payer (Payer is organization which takes care of financial and operational aspects (which include insurance plans, provider network) of providing health care to US citizens. Or refer to health care insurers.) and the regulator communities by illustrating the common data tracking needs, which highlights the need for coordinated data activities; and third, a proposed system architecture for scalable, multi-stakeholder data sharing. Next steps are briefly discussed.

**Conclusion:**

We present a system design for multiple stakeholders such as the payer, the regulator, the developer (drug manufacturer), and the healthcare provider to share data for their decision-making. The stakeholder community would benefit from collaboratively moving the system development proposal forward for efficient and cost-effective data sharing.

## Introduction

Data sharing among stakeholders in the drug development process is critical to their decision-making [1, 2]. For example, the Kefauver–Harris Amendments of 1962 have required pharmaceutical companies to report adverse events occurring with their drugs in the US market to regulators in order to make important safety decisions. In a recent study [2], it was reported that among the 222 novel therapeutics approved by the US Food and Drug Administration (FDA) from 2001 through 2010, 32% required safety updates following approval. Another stakeholder, the payer, needs treatment effectiveness information at the patient level in order to make payment decisions regarding value-based care. Post-marketing surveillance will be particularly important for gene and cell therapies, where treatment durability and long-term safety are both still unknown, therapies remain very expensive (for example, Zolgensma for SMA is priced at over $2 million), and the science continues to evolve rapidly. Yet, despite this need for sharing, the systems currently in place do not allow for easy data sharing. The reasons for this lack of interconnectivity stem fundamentally from the fact that each individual system was built to address a given stakeholder’s particular needs and priorities with accompanying legacy processes and infrastructure without consideration being given to standardization of data, procedures, and structure.

Post-marketing surveillance, in particular, will require carefully coordinated data sharing. Multiple data collection systems may create conflicting information in incompatible formats, and the incentives for sharing may not be aligned for the data owners. Even when the stakeholders do want to share data [3], the infrastructure may not be available or adequate [4]. Unguided, ad hoc creation of multiple systems for data collection and sharing leads to a complicated state with redundancies, other inefficiencies, and higher costs. Thus, there is considerable room for improvement in every aspect of data sharing.

We make three major contributions to the drug development and healthcare communities; first, a methodology for developing a multi-stakeholder data sharing system, with its focus on understanding high-level requirements that influence the design of the system architecture and accompanying technology enablers; second, the development of a realistic use case for long-term patient and therapy data tracking and sharing in the use of durable and potentially curative gene and cell therapies. Further, a bridge for the ‘awareness gap’ was found between the payer and the regulator communities by illustrating the common data tracking needs, which highlights the need for coordinated data activities that would benefit all stakeholders; and third, a proposed system architecture that would support scalable, multi-stakeholder data sharing, followed by a discussion of next steps.

## Methods

### A Systems Engineering Approach to Multi-stakeholder Data Sharing

There are many healthcare data initiatives aimed at accelerating data sharing, but their goals often focus on solving immediate problems with the latest technology (e.g., a cloud-based infrastructure, block chain, artificial intelligence, and so forth). These ad hoc efforts often provide modest improvements to existing processes rather than rethink the processes themselves, and thus technology-centric approaches might only achieve incremental improvements.

**Fig. 1:**
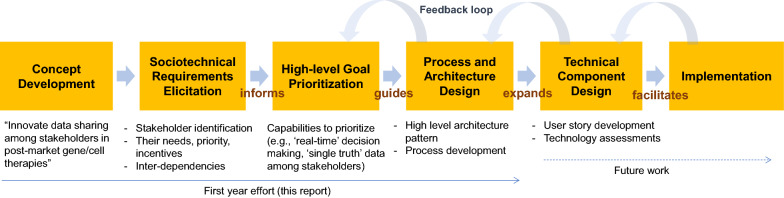
Our Systems Approach that Starts with High-Level Sociotechnical Requirements. Each Step is an Iterative and Incremental Process that can Inform the Next Step.

Instead, we take a systems engineering approach [5] to address the architectural and process level changes needed for a complex system design (Fig. 1). The architectural phase includes understanding of high-level user goals, needs, and other intangible qualities critical for anchoring the design of a complex system. These high-level requirements inform and guide the overall system design, which, in turn, address more granular, lower-level concerns, such as user experience, data ownership, and governance, and system-level requirements, such as performance, security, patient privacy protection, and access control. Our approach that together considers process, people, and technology [6] is needed for any large-scale data innovation, such as a digital transformation across subgroups within a large organization or the development of a new process and system used across multiple stakeholders.

Based on the authors’ extensive experiences in this domain, we considered the current processes in sharing data, high-level requirements, goals, desired capabilities, priorities, regulatory expectations, and pain points in prioritizing a number of high-level goals for a multi-stakeholder data sharing system (Fig. 3). The high-level goals, along with a realistic use case that we developed, informed the design of a scalable, neutral, and collaborative data sharing system. The authors’ backgrounds represent a range of stakeholder groups, including the pharmaceutical industry, the federal regulatory agency, the public and private payment systems, technology, and academia. Additionally, we elicited stakeholder input through interviews and meetings with twelve senior leaders from the above domains, including patient advocacy. Prototypical data sharing examples, such as a large pharmaceutical company’s requirements for regular safety updates to global regulatory agencies, were used to illustrate the inefficiencies and high cost of current processes and systems for data collection, management, submission, and sharing. We also consulted the broader NEWDIGS (NEW Drug Development ParadIGmS)^1^ initiative community through a presentation of the work at a ‘Design Lab’ (structured system design exercises core to the NEWDIGS methodology [7, 8]) whose 70 participants represented diverse range of stakeholder perspectives.

### Use Case Development

A realistic use case was developed in order to guide the design of the architecture for a multi-stakeholder data sharing system. We have chosen gene and cell therapies because they are unique in that they are permanently in place and not subject to the well-established pharmacokinetic principles of drug development, but rather are more concerned with the issues that arise with implantable medical devices that require long-term monitoring. Thus, we discuss the data tracking and sharing requirements for durable and potentially curative gene and cell therapies that are becoming increasingly critical as the number of such therapies is expected to greatly increase in the near future. While long-term patient tracking is not a new problem per se, we picked the particular case study for the following reasons: (1) The expected increase in the number of new products and new classes of products on the market create an urgent need for a multi-stakeholder data sharing system that would accelerate data tracking for safety and effectiveness decision-making. (2) The small number of currently available therapies and the small numbers of patients being treated by each of these therapies creates an ideal setting for a case study to test out. (3) The greater complexity involved in data tracking poses a greater challenge than most other therapies because patients and products would need to be tracked over longer periods of time, likely to be many years. (4) This further poses additional challenges in multi-stakeholder data sharing, as any given patient may change providers, insurers (payers), and/or states of residence, which can complicate data tracking and sharing due to interoperability issues of different systems and varying policies. Thus, a successfully structured multi-stakeholder data sharing system, such as the present proposal, could be easily adapted to other therapies such as Cystic fibrosis that share similar needs but with fewer complexities.

The contributions of the use case development are two-fold: (1) to help develop the details of a required data sharing system architecture and (2) to focus the drug development and healthcare communities’ attention on an emerging data problem that requires the joint communities’ concerted efforts to resolve it. As we discuss below, the particular use case for gene and cell therapies bridges an ‘awareness gap’ in the data tracking requirements that exist between the regulator and the payer groups. Tracking therapy *effectiveness* data and its patients is a topic for MIT NEWDIGS’s FoCUS (the Financing and Reimbursement of Cures in the USA) project [9], and tracking therapy *safety* data is required by the FDA as published in current guidance documents [10]. In Table 1, we provide a tabular summary of the stakeholders, issues and features of the system as a convenient reference to the particular details that are discussed in the following sections.

**Table 1:** Stakeholder Issues, Benefits, Disincentives, and System Design Needs.

Stakeholder	Issues	System Benefits	Disincentive	System Design Needs
Regulator	Need to track therapy safety and durability data over a prolong period of time (e.g., 15 years)	Obtain real-time safety and effectiveness data for making faster safety decisions Triage safety concerns based on pre-agreed metrics	Metrics-based decision-making may require additional safe guards	Examples of “successful” metrics-based decisions (e.g., the FoCUS initiative) Allow Regulator to easily define metrics and data requirements
Payer	Need to adjudicate contract based on the long-term performance of a therapy for a particular patient	Get complete effectiveness and safety data more quickly for making on-demand payment decisions for a particular patient	May want to go with incomplete, but simpler data tracking with current systems	Show clear benefits of extended data access and integration in payment decision
Developer (Pharma)	Desire to follow and submit therapy safety and effectiveness data in a cost-effective and accelerated way	Pool resources and use a common infrastructure, reduce cost and accelerate timing in regulatory submission Automation supported by the system will greatly reduce the time and cost	Desire to maintain authority over governance and data ownership Desire to protect own data such as clinical trials or registry data	Allow better control over own data Allow each company to customize private space boundaries and features
Provider	Relevant patient history is unavailable to improve patient care decisions	Obtain patient related information that may help enable better care	If the system is not streamlined, the cost for additional work to enter data may be prohibitive	Integration with existing EHR systems Understand what information is relevant for diagnosis
Patient	Desire to view patient data in a privacy protected way	Understand and control release of own clinical and product performance data	Implications for data release may not be easily understood Release control may be complex	Patient may desire more consumer friendly and customized access capability, and help in making data decisions

## Results and Discussion

We believe there are several highly desirable features needed for post-marketing data tracking and sharing. While not exhaustive, they do act as general design principles that can help develop a future data sharing system. Here, we briefly describe three infrequently discussed, but critical features that we believe are important for a post-marketing data sharing system.

**Fig. 2:**
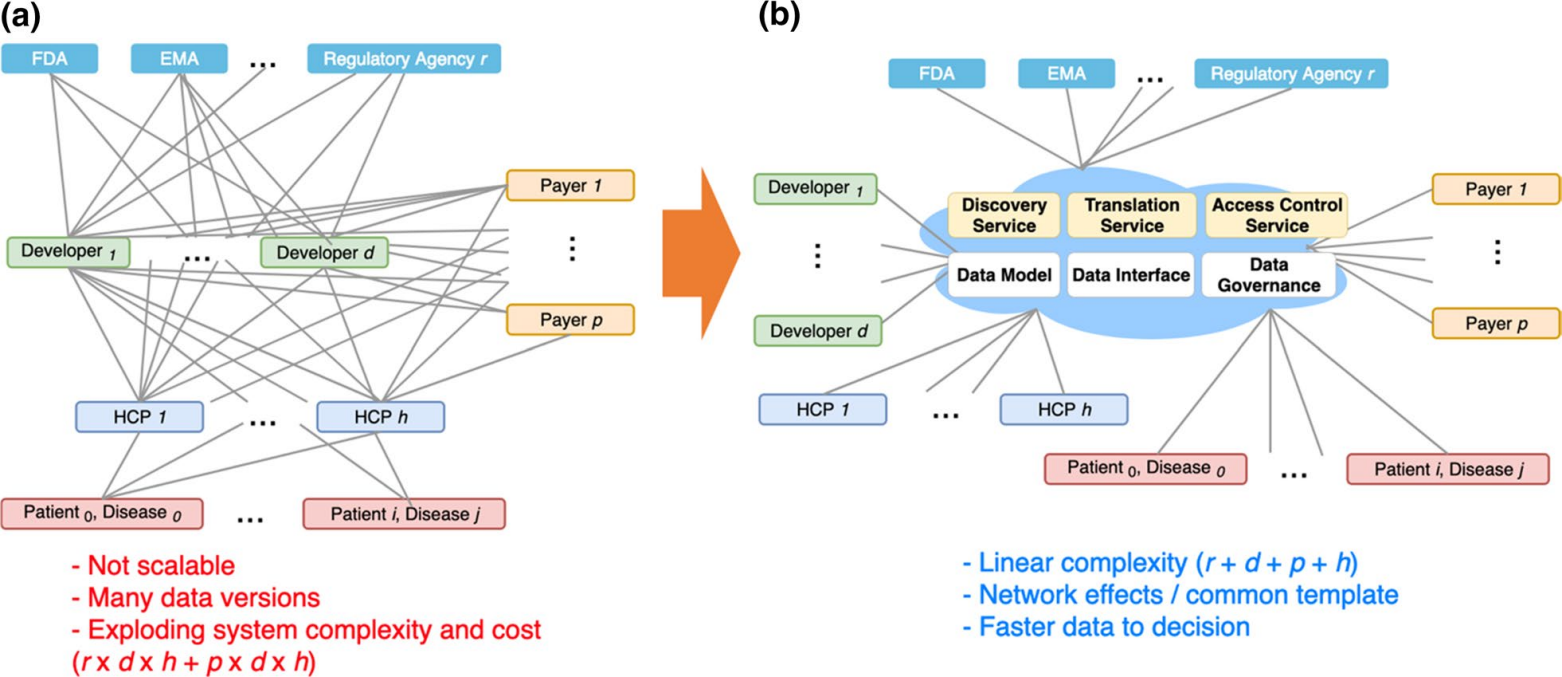
In the Absence of an Overall Guidance and Coordination, Each Developer, Payer, and Regulator Will Likely Create Propriety Systems to Track Data for Each Disease, Potentially Leading to Many Dependencies and Redundancies in Data Collection, Management, and Sharing (**a**). Instead, a ‘Virtually Centralized’ Infrastructure could be Set Up to Meet the Common Data Needs of Multiple Stakeholders, with Commonly Provided Services and Resources that the Members Could Utilize in Working with Others (**b**).

- **‘Real-time’ decision-making** we define ‘real-time’ as, however, fast is necessary to enable a stakeholder to make a decision in a timely manner for their own needs. For example, for the regulator who wants to know when significant adverse events occur, ‘real-time’ could mean days or weeks after the event occurs. ‘Real-time’ requirements and existing technology are often not aligned with one another. However, they are mutually influencing, in that a policy may be set in a certain way because of limitations of technology, but technological advances may eventually lead to changes in policy. It is important to identify all of the stakeholder data interdependencies, their latencies, and the ‘slowest in the link’ in order to design the system and process that would facilitate real-time decision-making.
  One related concept is *data triaging* when there is a large volume of data to process. Prioritizing and down selecting prominent data nuggets from the rest would be critical in situations such as adverse event reports that the regulator must review. Another related concept is *metrics-based* reporting. The process of sharing full patient data may be impaired because of heightened policy and technology barriers, or may not be desired in some cases. Instead, pre-agreed upon metrics with automated, and auditable features could be developed to facilitate stakeholder decision-making. As described in the system architecture (Fig. 2b) and discussed in the ‘Dynamic Dossier in the Cloud’ section, the envisioned system will help enable “real-time” and metrics-based data sharing and access.
- **‘Single truth’** data are frequently collected multiple times by different users for varying purposes. The systems that store and process these data are siloed and not interoperable, inevitably leading to different versions of the ‘truth,’ which becomes problematic when multiple stakeholders individually make important decisions that affect one another based on conflicting and inconsistent data. Moreover, much of the data is related to a patient and conflicting data about the patient may have major impact on his/her life. A patient-centered approach in storing, sharing, and using data would be needed, and it would be critical to create and to preserve a reference or master data set in a data sharing system.
- **Scalability** (in the sense of membership) we are less concerned about the data size or data storage requirements than about the number of varying types of data and user entities joining the data sharing activities. Each new system or stakeholder may bring a different format or data type that has other system requirements, and the system would not be scalable if everyone is required to make a change in the way their system works with other systems in order to be compatible. There is a push for more interoperable and standard-based solutions in the drug development domain, and this should continue. The system should also support scalable addition of disparate data formats and contents through common components and standards the community could utilize.

**Fig. 3:**
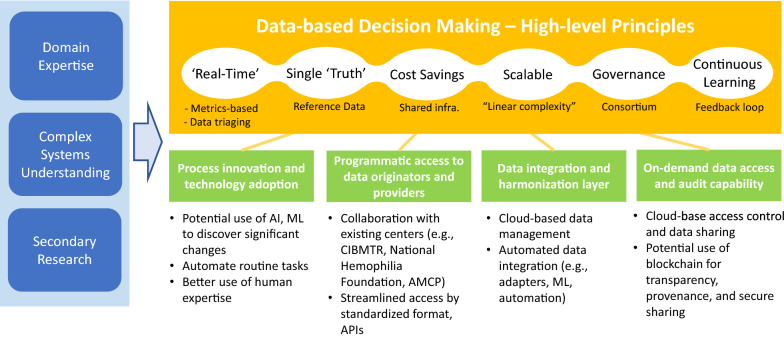
Capability Requirements of the Dynamic Dossier in the Cloud Informed by Domain Expertise, Systems Understanding, and Secondary Research. Both Process Innovation and Technology Adoption are Necessary to Enable the High-Level Principles.

For these and other high-level design principles that we believe are important, we lay out in Fig. 3 the next-level technology adoption and process change requirements that would be needed. There are other system-level issues such as protecting confidentiality in data sharing between different stakeholders, including drug manufacturers and regulators. Pharmaceutical companies have been required to report adverse events to FDA since the 1960s following passage of the Kefauver–Harris Drug Amendments of 1962. In the intervening decades, companies have expanded their sources of safety information beyond ad hoc receipt of adverse event reports, that is, Individual Case Safety Reports, from healthcare professionals and consumers to include (1) systematic, periodic, usually weekly, surveillance of the world's digitized literature for independent investigator study results and for case reports; (2) data-mining activities conducted on large-scale safety databases, both company managed and regulatory based; (3) patient registries that may be sponsored by academic or nonprofit institutions; (4) post-approval safety studies that may be recommended or required by regulatory agencies; and (5) company manufacturing product complaints that may be associated with adverse events [11]. In recent decades, companies and FDA have developed sophisticated data-mining capabilities to detect potential drug-adverse event associations in their data bases. Further, FDA conducts routine data-mining on its FDA Adverse Event Report System (FAERS) and posts their results quarterly, consistent with FDA Amendments Act of 2007. All information is anonymized, documented, access-controlled, encrypted, and secured. Companies and FDA have routinely used these and other sources of safety information to update company product labels for decades. The proposed system is envisioned to provide a consistent and automated way to use extant technologies such as access control, secure multi-party computation using encryption, deidentification, and others, in order to lower cognitive investment and associated monetary costs and to mitigate regulatory and security risks for participating stakeholders to share data. Other system implementation concerns also exist including security, encryption, privacy, performance, availability, user interface ease-of-use, and interoperability, as well as policy issues, including HIPAA [12] and GDPR [13].

We next discuss the use case we developed for a data sharing system whose design would support the prioritized goals discussed earlier. The use case was developed around emerging gene and cell therapies whose safety and effectiveness profiles are of increasing importance. The payer’s and the regulator’s concerns around gene and cell therapies provide both background information and the data tracking requirements that inform the design of a data sharing system. We discuss each in turn in the subsequent sections.

### The FoCUS Project on Effectiveness Data Tracking

The Financing and Reimbursement of Cures in the US (FoCUS) Project [9] was launched in 2016 by MIT NEWDIGS with the aim of delivering an understanding of the challenges and impact of innovative cell and gene therapies on the UShealthcare ecosystem. When examining the effectiveness of a potentially curative therapy, it has become evident that data collection and patient mobility are among the primary issues identified.

Current challenges for the stakeholders include the following: identifying which stakeholder will be collecting the data; what metrics will be measured; what data sources will be available and considered reliable; where data sources are located; and patient availability to provide data over time. For instance, in order to administer a multi-year value-based contract, data capability enablers (for example, the Center for International Blood and Marrow Transplant Research (CIBMTR) [14] for blood disorders from hemophilia to sickle cell anemia and beta thalassemia) would need to be in place in order to affirm that performance metrics are met. Further, if a patient were to uproot and change Health Plans in the middle of a multi-year value-based contract it would cause considerable complications for data tracking. Sharing patient performance data among the involved parties in performance-based agreements (especially developers and subsequent payers) often requires additional hurdles due to HIPAA regulations that did not contemplate this sort of agreement. Thus, amending HIPAA regulations may be needed to ensure that those investing in patient health through long-term performance-based agreements can access needed, relevant data. In practice, however, patients may waive certain aspects of these statutes in order to provide on-going data to drug developers and providers as part of (1) improving care and (2) ensuring that they get the most up-to-date care possible. In this case, however, safeguards need to be set up in order to protect the patients and allow them to have a control over their own data. While many privacy challenges of patient tracking exist [15], to address the policy changes necessary is beyond the scope of this paper. We believe the proposed system where multi-stakeholders’ needs (including the patients’) can be articulated would better highlight what changes would be needed.

The FoCUS Project has proposed multiple precision financing solutions to help address the financial challenges associated with implementing gene and cell therapies. These new methods of payment require access to data in a timely and scalable way in order to make decisions regarding treatment effectiveness and whether continued payment is merited. Thus, a method of collecting and tracking outcomes data in gene and cell therapies from different data sources such as various hospitals and treatment centers to support value-based payment decision-making for the payer.

### FDA Requirements on Safety Data Tracking

While only a small number of gene and cell therapies are approved in 2019, FDA estimates that it will receive approximately 200 Investigational New Drug applications in 2020 for cell-based or directly administered gene therapy INDs, and that by 2025, it will be approving 10 to 20 cell-based and gene therapy products per year [16]. To support the advancement of these product technologies into effective therapies, new policy guidance documents were introduced in 2019. FDA has signaled that it intends to use the regenerative medicine advanced therapy (RMAT) designation and accelerated approval as the key regulatory pathway for these products to obtain market approval, particularly for products intended to address unmet medical needs, and to continue to focus attention on individual products after approval through follow-up studies. These mechanisms will help to answer questions about durability of clinical benefit and the potential for rare adverse reactions due to off-target effects.

FDA has published *Guidance for Industry* [17] regarding Expedited Programs for Regenerative Medicine Therapies for Serious Conditions (February 2019) to provide sponsors with regulatory recommendations on expedited development of, and FDA’s review perspective on, products meeting the definition of ‘regenerative medicine advanced therapy’ (RMAT) [10]. RMAT designation entitles the holder to the benefits of fast track and breakthrough therapy designation programs. Depending upon certain features of the intended disease and the goal of therapy, the product may also be eligible for priority review designation and accelerated approval. Accelerated approval designation has implications for post-approval adverse event reporting. Sponsors of such drugs may be required to conduct post-approval confirmatory studies to verify and describe the anticipated effects of their products on irreversible morbidity and mortality or other clinical benefit.

Any system intended to track safety reporting for these products must be flexible enough to build in provisions for patient registries and other sources of real-world evidence. As the therapies have potential risks of delayed adverse events, a long-term safety and patient tracking is desirable.

### The Need for Coordinated Data Collection, Tracking, and Sharing

**Fig. 4:**
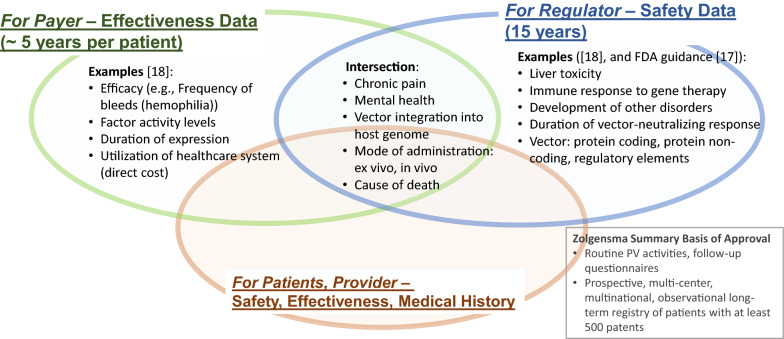
A Notional Diagram that Illustrates an Overlap Among Data Requirements from Stakeholders. Note that the Venn Diagram is not Meant to Show Where the Division is, Rather It is Notionally Depicting There is a Common Data Concern.

We note the differing needs of payer and regulator: (1) the payer would need to track a therapy’s effectiveness data on individual patients in order to make a payment decision in a multi-year value-based contract, and (2) the regulator would require long-term tracking (15 years [17]) of the safety data. Figure 4 shows a notional diagram that while the payer and regulator each has a different goal in tracking data, many of the data metrics align and would be of interest to both communities. It is likely, for example, that the regulator’s safety data would influence the payer’s decision on the effectiveness of a therapy, and likewise, that the effectiveness data would also be a factor considered by the regulator in a benefit-risk analysis. Overlapping data tracking requirements for the payer and the regulator implies that coordinated data collection would have the potential to mitigate the risks of data conflicts and to reduce the high costs incurred by collecting the same data multiple times, which can lead to inconsistencies if not careful. Patients and providers would also benefit from the collected data in making a medical decision about therapies. Thus, the larger stakeholder communities would benefit from enhanced collaboration in data collection, storage, formatting, and sharing, and coordinated access to data would be enabled in a consistent way.

**Fig. 5:**
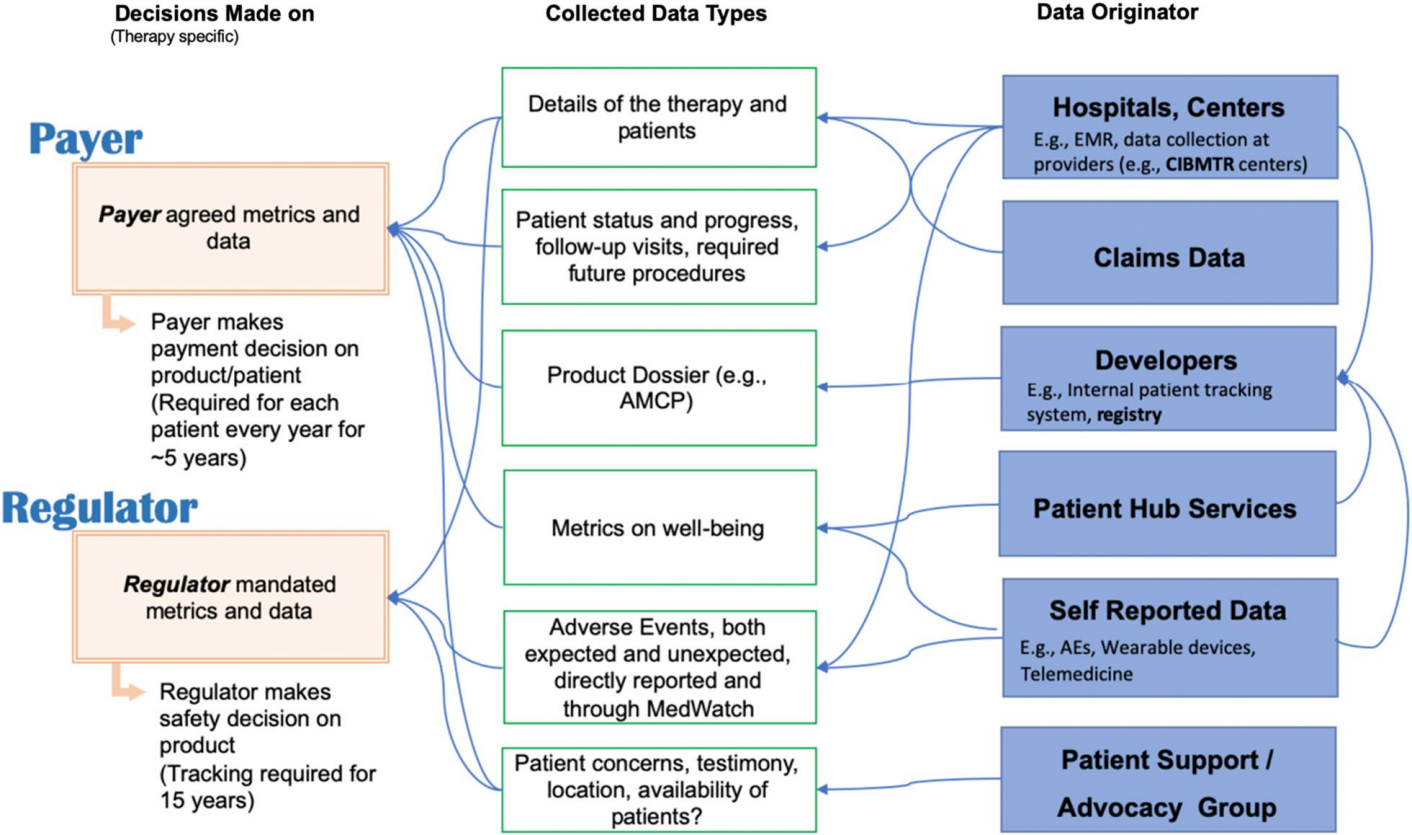
A Notional Diagram Illustrating the Complexity in Data Collection and Sharing.

The Venn Diagram in Fig. 4 shows the inherent complexity in collecting and sharing data for the gene and cell therapy use case as addressed by prior work [17, 18], and Fig. 5 further exemplifies the need for coordinating data activities. The data elements shown in Fig. 4 could be considered types of ‘decision metrics’ with which each stakeholder measures effectiveness and safety of a therapy. The raw data needed for each metric may come from any of several data originators as shown in example Fig. 5. Already, some redundancies and multiple pathways exist that could lead to multiple data collection efforts and potential conflicts in data versions. Note that this is when only *one* regulator, *one* payer, and *one* product are considered. In reality, each developer, in the absence of a high-level guidance or established, shared infrastructure, would likely create a custom solution for its own particular product, which is probably the most efficient way to operate when considering a situation in isolation. However, it quickly becomes an exponentially complex problem for all stakeholders when there are multiple products, developers, payers, and regulators that must work together but for which each pair, in fact, develops its own platform, data format, and data submission workflows (as illustrated in Fig. 2a). The greater complexity and higher cost with potentially more conflicted data make this situation very undesirable and even harmful.

The regulator and the payer may disregard the problem assuming it is the developer’s responsibility to submit the necessary data for safety and effectiveness reviews in a timely manner. However, the greater complexity and higher cost of data collection, management, and sharing may keep developers from submitting data in an effective and efficient way, which, in turn, may greatly impact the regulator’s ability to enforce safety policies and the payer’s ability to make timely payment decisions. In fact, the regulator and the payer are in a unique collaborative position to set guidance and enforce the system and process design that would clearly benefit every stakeholder, including the regulator and the payer themselves. We discuss what such a system and process would look like in the next section.

### The Dynamic Dossier in the Cloud

Instead of each member of the triad, that is, developer, regulator, and payer, creating a custom solution for each therapeutic product, we propose a neutral, intermediary, and collaborative environment that we call ‘the Dynamic Dossier in the Cloud’ that would facilitate data sharing and submission for multiple stakeholders (Fig. 2b). Taking advantage of *common interfaces* and *data models*, this approach would greatly simplify the architecture of a multi-stakeholder data sharing system, and the complexity would only increase in a linear and manageable fashion as more stakeholders join. An effective design for a complex system requires some degree of conformity to common sets of interfaces and data models. Although individual stakeholders would bring multiple, different interfaces and models, it would be possible to iteratively create a few localized standard interfaces and data models that would work for identified subgroups that need immediate collaboration (more discussion below).

The Dynamic Dossier would also provide reusable components and services that would facilitate data discovery, exchange, and, ultimately decision-making. It would enable positive network effects in which the value of the overall system and the benefits to each individual stakeholder would grow as more stakeholders join. For example, as one’s data were translated to a common data model, it could be used by an increasing number of stakeholders as the number of the participants grows.

### Architecture and Components of the Dynamic Dossier System

At a very high conceptual level, this ‘virtually centralized’ architecture connects all participating stakeholders; however, the system must still address several very difficult challenges of designing the technical components in order to enable such a highly collaborative environment. We touch upon the high-level architectural and component recommendations for the Dynamic Dossier system. While the technical solutions may already exist for particular problems, thus possibly suggesting that this may be a readily solvable problem, the societal and human factors are similarly multiple and potentially complex, which often lead to failures of multi-stakeholder systems. For example, there may be unforeseen patient privacy issues in releasing and controlling sensitive data, security risks in storing data in a shared environment, or a mal-aligned incentive structure. Thus, we believe a careful architectural and system-level design approach is critical in order to create a truly working multi-stakeholder system, as discussed above in the sociotechnical systems section. The technical choices discussed below would inform the future implementation of the system, about which further discussions and consensus among the stakeholders would be needed to develop the next level of detail.

### Unique Patient ID

Part of the difficulty in the long-term tracking of patients is that patients may move between different payers, providers, systems, and jurisdictions. Each system and organization may employ its own patient ID scheme, such that matching patients across different systems is impossible unless there is a way of mapping all of the different IDs or creating another unique patient ID. Issues of and approaches to creating a unique patient ID have been much discussed, for example, [19]. The Dynamic Dossier in the Cloud system is envisioned to provide a unique patient ID scheme together with a translator service that would map the patient IDs of existing systems involved in tracking gene and cell therapy patients. Further discussion among the involved stakeholders would be needed for specific implementation.

### Hybrid Cloud-Based Infrastructure

At a high level, the environment is technology agnostic, but practicality suggests a Cloud-based infrastructure [20] and industry leaders are already moving toward it [21]. Although the Cloud by itself promotes neither more nor less data interoperability, nor necessarily faster data exchange, its characteristics are ideal for multi-party co-ownership: (1) access to consistent, state-of-the-art security that reduces the importance of the ‘weakest link’ among stakeholder systems; (2) scalability, in the computing and data size sense (however, membership scalability as described in Fig. 2b is achieved by the architectural design, not by the use of the Cloud); and (3) lower operational costs. The Dynamic Dossier in the Cloud is envisioned to provide several services and components that would be available to stakeholders in order to perform their necessary data activities as discussed below. A hybrid cloud approach would also permit interfaces between private, proprietary clouds that individual stakeholders own to keep their data and a publicly shared cloud where the stakeholders release certain data in a controlled manner. Security and privacy issues, such as those needed to be compliant with HIPAA, must be thoroughly addressed to protect sensitive data, especially for patients who often do not have a say in the design and control of such a system.

### Standard Data Models and Interfaces

Interoperability issues arise for many reasons, including systems independently developed in silos, syntactic (data formats and structures) and semantic (the meaning of terms) differences across domain and systems, and explicit barriers created in order to protect intellectual properties, and the lack of guiding standard. A greater degree of interoperability is needed at multiple levels such as the policy, access, document, and data.

At the policy level, for example, the new Health and Human Services rules of implementing interoperability and patient access of the 21st Century Cures Act set the environment for encouraging interoperability. Other standards such as the Fast Healthcare Interoperability Resources (FHIR) allow building and accessing composite documents in a standardized way, and the Consolidated Clinical Document Architecture (C-CDA) provides a consistent document format, albeit static. At the lower data level, more data and interface conformity are needed in order to achieve membership scalability in a data sharing system. For example, the FDA’s Sentinel initiative [22] enables working with multiple healthcare data systems through a common data model [23] to which the data owners conform their data. This works well when the distributed data sources contain similar types of data, the groups are more homogeneous, and the data model is relatively simple. In our case, however, the number and types of disparate systems, stakeholders, and their use cases would be too high for everyone to conform to one common data model. Systems of different subgroups of stakeholders may need to interact for a specific therapeutic product, and there would be many different therapies that need tracking. Common problems include the following:

- Syntactic incompatibility: Data format differences among the systems
- Semantic incompatibility: Inconsistencies in the usage of data
  - The problem of polysemy: Stakeholders may use the same term to describe different concepts
  - The problem of synonymy: Stakeholders may describe the same concept with different terms
- Data model non-alignment: Often, it may be too difficult or undesirable for everyone to conform to one common data model due to the subtle differences in the stakeholder domains

Instead of a simple common data model, a more appropriate approach would be supporting multiple (evolving) models using a flexible encoding mechanism, such as an ontology. An ontology [24] can describe important concepts and their relationship and is extensible both with new and existing ontologies. Some standards [25] are already in an ontology, and many programming languages support the creation and interfacing with it [26]. Ontology could help resolve the issues of syntactic and semantic incompatibilities, as well as model alignment issues. Metrics that different stakeholders are interested in could be described in an ontology, allowing consistent and automated communication.

But an ontology is only a tool to organize concepts together; the stakeholders would still need to create common data models to be described in the ontology through governance, and we propose a layered and iterative approach.

- If we define the stakeholder *roles* for a specific activity, we could identify their roles as either *data providers* or *data consumers*. Note a stakeholder could be both a provider and a consumer of data, as some decisions are made not just based on their own data, but with someone else’s too.
- For example, hospitals would be data providers as they would collect patient data and make them available to other consumers such as the payer. They would also be consumers if some patient data came from other sources, for example, patient worn sensor devices. A developer could be considered both a consumer and provider as they may get data from registries and submit them to the regulator.
- Subgroups of data providers for one or more related therapies could collaboratively create a common data model in an ontology for their use case, with the input from their direct data consumers to meet their needs.
- Since ontologies are extensible, the few emerging ontologies could be combined later to reduce redundancies. Creation of a common ontology and extensions to it would be especially useful when many slightly different submission data need to be prepared.

Scenario or use-case-based approaches to detailing out workflows would help identify the involved stakeholders, their systems, and the necessary encoding schema for a common ontology.

The system is envisioned to be highly customizable to a particular stakeholder’s data sharing needs. Each stakeholder would determine how much data to share and in what manner. Although initially a custom “adapter” would need to be built for individual systems currently in use, the envisioned goal is a de facto standard among closely collaborating stakeholders that would organically form over time and that new systems would conform to the interfaces that the stakeholder communities collaboratively come up with. One reason for data incompatibility is there often is no guiding principle or standard that individual system developer could conform to, and it is envisioned our proposed system would create an environment where the community creates such a standard that could be discovered by others.

We can imagine certain data may be more difficult to collect and track; for example, patient data collected by wearable devices would be harder to consistently track due to the incompatibility among the device manufacturers. Again, our proposed system would help in this case by providing a standard and a common place where data could be securely shared and discovered, with proper control and privacy protection measures.

We believe a top-down approach of creating one set of common data for all the possible use cases would be unachievable for a sufficiently complex system. Instead, it would preferable to start with small examples of data models created in an incremental, iterative, and collaborative way in a system environment that supports such growth. A multi-stakeholder data governance model should be implemented in order to guide the ontology creation or combination activities. The Dynamic Dossier system is envisioned to support such operations with technology components, services, and a multi-stakeholder consortium that guides the system’s direction. FDA’s Sentinel System offers a template for how to develop a step-wise approach to a workable project that was accomplished through the quinquennial review and funding initiated with the FDA Amendments Act of 2007 [27].

### Federated and Distributed Data Ownership

It is unrealistic to pursue a truly centralized system where every stakeholder puts their own data into a common place, even with proper access control and security measures. Instead, a federated learning and data architecture [28] would be a better approach that would allow each data owner to keep and control the type and amount of data release and access to it. The FDA’s Sentinel system, for example, allows each healthcare data owner to maintain ownership and control of the response to a query. The data owner would be able to control who could receive which subset of data through access control. Multiple stakeholders would also be able to run an analysis directly on one another’s data without sharing them through a secure multi-party computation [29]. A hybrid model could be considered where most of the data reside on the stakeholders’ own systems and only shareable data are put into access-controlled central location.

Consistent data models, interfaces, and access would benefit all of the stakeholders that use the system to share data among them. One additional benefit, when a system such as the Dynamic Dossier in the Cloud is established and used by many, is the use of its model and interfaces as a ‘template’ for each individual company’s internal digital transformation activities. Many companies go through digitalization and automation innovations in order to streamline their internal processes and systems, but may end up with models incompatible with external entities as innovations happen within the boundaries of individual companies. An externally established system, such as the Dynamic Dossier in the Cloud, could act as a guiding system, allowing companies to inform their digital transformation to be more compatible with the rest of the world.

Finally, there are the “next steps” that would be needed to implement such a project: the building of consensus among stakeholders to marshal the political forces needed to back and to fund the project. As mentioned above, FDA’s Sentinel Project offers a successful approach to achieving this goal.

## Conclusions

In this paper, we have discussed the need for a Systems approach to the design of a multi-stakeholder data sharing system that starts with high-level stakeholder concerns and creates prioritized goals that influence the system design. The use case we developed illustrates the emerging need for *coordinated* data tracking for novel gene and cell therapies. The design for a scalable and collaborative system has been proposed in order to provide a viable solution to a complex problem.

Further work is needed to bring the high-level concepts we have developed closer to actual implementation. For example, stakeholder incentives and needs for each of the critical features of the system would need to be better understood through design exercises (See Table 1 for high-level examples of design needs), as well as detailing out technical component designs discussed in this paper. Most importantly, the success of a multi-stakeholder system such as the Dynamic Dossier in the Cloud would only be achievable if the members of the stakeholder community come together and collaboratively move it forward.

## Footnotes

1 https://newdigs.mit.edu/.
